# Fusion of Bacterial Flagellin to a Dendritic Cell-Targeting αCD40 Antibody Construct Coupled With Viral or Leukemia-Specific Antigens Enhances Dendritic Cell Maturation and Activates Peptide-Responsive T Cells

**DOI:** 10.3389/fimmu.2020.602802

**Published:** 2020-11-12

**Authors:** Saskia Schmitt, Siret Tahk, Alina Lohner, Gerulf Hänel, Andreas Maiser, Martina Hauke, Lubna Patel, Maurine Rothe, Christine Josenhans, Heinrich Leonhardt, Marieke Griffioen, Katrin Deiser, Nadja C. Fenn, Karl-Peter Hopfner, Marion Subklewe

**Affiliations:** ^1^ Gene Center and Department of Biochemistry, Ludwig Maximilians University Munich, Munich, Germany; ^2^ Department of Medicine III, University Hospital, Ludwig Maximilians University Munich, Munich, Germany; ^3^ Gene Center Munich, Laboratory for Translational Cancer Immunology, Ludwig Maximilians University Munich, Munich, Germany; ^4^ Department of Biology II, Center for Integrated Protein Science, Ludwig Maximilians University Munich, Munich, Germany; ^5^ Max von Pettenkofer Institute, Ludwig Maximilians University Munich, Munich, Germany; ^6^ German Center of Infection Research, DZIF, Munich, Germany; ^7^ Department of Hematology, Leiden University Medical Center, Leiden, Netherlands; ^8^ German Cancer Consortium (DKTK) and German Cancer Research Center (DKFZ), Heidelberg, Germany

**Keywords:** dendritic cell, flagellin, neoantigen, vaccine, antibody, acute myeloid leukemia

## Abstract

Conventional dendritic cell (DC) vaccine strategies, in which DCs are loaded with antigens *ex vivo*, suffer biological issues such as impaired DC migration capacity and laborious GMP production procedures. In a promising alternative, antigens are targeted to DC-associated endocytic receptors *in vivo* with antibody–antigen conjugates co-administered with toll-like receptor (TLR) agonists as adjuvants. To combine the potential advantages of *in vivo* targeting of DCs with those of conjugated TLR agonists, we generated a multifunctional antibody construct integrating the DC-specific delivery of viral- or tumor-associated antigens and DC activation by TLR ligation in one molecule. We validated its functionality *in vitro* and determined if TLR ligation might improve the efficacy of such a molecule. In proof-of-principle studies, an αCD40 antibody containing a CMV pp65-derived peptide as an antigen domain (αCD40^CMV^) was genetically fused to the TLR5-binding D0/D1 domain of bacterial flagellin (αCD40.Flg^CMV^). The analysis of surface maturation markers on immature DCs revealed that fusion of flagellin to αCD40^CMV^ highly increased DC maturation (3.4-fold elevation of CD80 expression compared to αCD40^CMV^ alone) by specifically interacting with TLR5. Immature DCs loaded with αCD40.Flg^CMV^ induced significantly higher CMV_NLV_-specific T cell activation and proliferation compared to αCD40^CMV^ in co-culture experiments with allogeneic and autologous T cells (1.8-fold increase in % IFN-γ/TNF-α^+^ CD8^+^ T cells and 3.9-fold increase in % CMV_NLV_-specific dextramer^+^ CD8^+^ T cells). More importantly, we confirmed the beneficial effects of flagellin-dependent DC stimulation using a tumor-specific neoantigen as the antigen domain. Specifically, the acute myeloid leukemia (AML)-specific mutated NPM1 (mNPM1)-derived neoantigen CLAVEEVSL was delivered to DCs in the form of αCD40^mNPM1^ and αCD40.Flg^mNPM1^ antibody constructs, making this study the first to investigate mNPM1 in a DC vaccination context. Again, αCD40.Flg^mNPM1^-loaded DCs more potently activated allogeneic mNPM1_CLA_-specific T cells compared to αCD40^mNPM1^. These *in vitro* results confirmed the functionality of our multifunctional antibody construct and demonstrated that TLR5 ligation improved the efficacy of the molecule. Future mouse studies are required to examine the T cell-activating potential of αCD40.Flg^mNPM1^ after targeting of dendritic cells *in vivo* using AML xenograft models.

## Introduction

Dendritic cells (DCs) play a key role at the interface between innate and adaptive immunity, and therefore hold potential for use in the immunotherapy of diseases such as cancer. In particular, the high capacity of DCs for processing and presenting antigens makes them suitable candidates for manipulating with antigens of choice in a vaccine approach. The most common strategy is to load DCs *ex vivo* with major histocompatibility complex (MHC)-binding peptides. This has been investigated in numerous clinical trials in different cancer entities, which have so far shown safety and feasibility, but often lack efficacy (1, 2). Improvements have been achieved with the development of personalized neoantigen-based DC vaccines, which elicited potent neoantigen-specific T cell responses with remarkable efficacy in melanoma patients (3–5). However, this type of DC vaccination features drawbacks. The *ex vivo* engineering and GMP production of DCs is costly and labor-intensive, and standardization is difficult as vaccines are generated individually for each patient (6). In addition, the efficacy of these vaccines can be limited by inefficient migration of administered DCs to the lymph nodes, wherein DCs activate antigen-specific T cells (7).

An alternative approach consists of delivering an antigen to target DCs *in vivo* using an antibody–antigen fusion construct. Such vaccines can be applied to a larger patient cohort and thus be manufactured on a larger scale. More importantly, this technique has biological advantages as it exploits the intricate migratory capacity of DCs *in situ* and directly activates natural DC subsets at multiple sites *in vivo* thereby producing a more physiological DC maturation (8, 9). Although clinical data are still scarce, *in vivo* DC vaccination is considered a promising strategy for eliciting strong and sustained T cell responses (2, 10, 11).

Different DC surface receptors have been proposed as targets for *in vivo* DC vaccines. These differ widely in their expression levels, intracellular trafficking pathways and antigen presentation capacity. Among those, CD40 is of high therapeutic interest. Indeed, previous pre-clinical studies showed that the delivery of antigens to DCs by CD40-targeting antibodies was more efficient in eliciting MHC-I cross-presentation and inducing CD8^+^ T cell responses compared to other receptors such as Dec205 (12–14). The 48 kDa type I transmembrane protein CD40 is a critical mediator of immune cell communication, for example, by initiating T cell priming, and is a costimulatory surface receptor of the tumor necrosis factor receptor (TNFR) family (15). Importantly, agonistic αCD40 antibodies not only facilitate DC-targeting, but also exhibit adjuvant function by inducing CD40 signaling to transduce an intrinsic stimulatory signal to DCs.

The use of adjuvants is particularly important for DC vaccination. At steady state, immature DCs tend to induce tolerogenic T cell responses (16). However, DCs mature and upregulate co-stimulatory molecules in the presence of adjuvants, thereby enhancing cross-talk with T cells. In addition to CD40-activating agents, ligands for toll-like receptors (TLRs) are commonly used that potently activate innate immunity and are critical for optimizing T cell responses (17). If a single adjuvant is insufficient for DC activation, using a combination of adjuvants has been proposed, especially for targeting multiple intracellular signaling pathways (18, 19). Ahonen et al. have shown that co-administration of CD40 activators together with various TLR agonists induced higher antigen-specific CD8^+^ T cell responses than either agonist alone, demonstrating the synergy between TLR-derived stimuli and the CD40 pathway (20). TLR3 or TLR7/8 agonists are being used as adjuvant drugs in various clinical trials, and TLR5 agonists have been recently investigated (21, 22). As TLR5 detects bacterial flagellin—the protein that polymerizes to form flagella—flagellin or constitutive domains can be used as activators (23). A clinically advanced example is entolimod, a pharmacologically optimized truncated version of flagellin from *Salmonella enterica* serovar Dublin, which retains the two domains (D0/D1) essential for TLR5 binding (24). Entolimod was initially established as a potential treatment for lethal radiation exposure in patients with advanced solid tumors due to its tissue protective activity (24–26). It is also reported to elicit direct anti-tumoral effects through activation of the NK–DC–CD8^+^ T cell axis and demonstrated anti-metastatic activity through immune stimulatory mechanisms involving NK cells (26–29). More recently, entolimod is investigated as a vaccine adjuvant by its developing company Cleveland BioLabs.

Importantly, not only does the nature and optimal combination of adjuvants determine the outcome of DC vaccination strategies but also the administration regimen. In practice, adjuvants are typically co-administered with DC-targeting antibodies, which has been shown to improve the therapeutic efficacy of DC-targeted vaccines. However, the use of soluble adjuvants can lead to antigen-independent activation of bystander immune cells, which carries the risk of adverse events occuring, such as cytokine release or autoimmunity (30, 31). Indeed, targeted delivery of TLR agonists to DCs was not only associated with a reduced serum cytokine release and related toxicity, but also reduced their dose requirement by 100-fold (31). Furthermore, co-delivery of antigen and adjuvant into the same antigen-presenting cell (APC), for example, in the form of antigen–adjuvant conjugates, resulted in superior cross-presentation and peptide-specific T cell activation compared to administration of separate molecule *in vivo* (31–34).

To combine the benefits of *in vivo* co-delivery of a TLR agonists and an antigen, we generated novel multifunctional antibody constructs (αCD40.Flg^CMV^ and αCD40.Flg^mNPM1^) to target viral- and tumor-specific antigens to DCs *via* an αCD40 antibody. These simultaneously activate TLR5 with a genetically fused truncated entolimod-like flagellin domain (Flg). As antigen domain, we either fused a cytomegalovirus (CMV)-specific epitope or a neoantigen derived from mutated NPM1 (mNPM1), which occurs in 30–35% of acute myeloid leukemia (AML) (35, 36). We addressed the question of whether TLR5 ligation ameliorates the *in vitro* efficacy of αCD40^CMV^ and αCD40^mNPM1^ by evaluating these molecules based on their binding specificity, DC maturation and induction of viral- and tumor-specific T cell responses. Since the addition of a flagellin domain was indeed able to enhance DC maturation and to increase both CMV- and mNPM1-specific T cell responses compared to αCD40^CMV^ or αCD40^mNPM1^ alone, we consider this novel antibody–TLR agonist fusion format a promising therapeutic approach worthy of investigation in *in vivo* studies.

## Material and Methods

### Expression and Purification

The variable domains of αCD40 were derived from the IgG2 antibody CP-870,893 (selicrelumab, clone 21.4.1., Hoffman-La Roche) (37, 38). The αHer2 non-specific binding control contained variable domains from the 4D5-8 clone (trastuzumab, Hoffman-La Roche) (39). To produce IgG1 molecules, we cloned the coding sequences for both antibodies into the mammalian expression vectors pFUSE2-CLIg-hk and pFUSE-CHIg-hG1 (InvivoGen). P329G, L234A, and L235A (PGLALA) mutations were inserted to silence the Fc function (40). The flagellin D0/D1 domain (Flg), with a GSGGG linker introduced in place of D2/D3 of full-length flagellin, was cloned from genomic DNA of *Salmonella enterica* Typhimurium strain SL1344. The antigen domain consisted of either a sequence of the cytomegalovirus (CMV)-specific pp65 protein (CMV_487–508_)****including the HLA-A*02:01-restricted CMV_495–503_ epitope (NLVPMVATV), or the mutated NPM1 derived mNPM1_277–298_ protein that comprises the HLA-A*02:01-restricted mNPM1_288–296_ neoepitope (CLAVEEVSL). The CMV_487–508_ domain is hereafter abbreviated as CMV, and the mNPM1_277–298_ domain as mNPM1; the epitope peptides are denoted CMV_NLV_ and mNPM1_CLA_, respectively. The antigen domains were cloned to the C terminus of the light chain and flagellin to the heavy chain, both separated by (G_4_S)_4_ linkers. As a control, an Fc–flagellin fusion was generated, which consisted of an IgG1 Fc region followed by a (G_4_S)_4_ linker and flagellin on the C terminus.

All molecules were produced over 5 days in Expi293F cells (Thermo Fisher Scientific) according to the manufacturer’s instructions. For purification, protein A affinity chromatography (nProtein A Sepharose 4 Fast Flow, Thermo Fisher Scientific) was performed followed by size-exclusion chromatography (SEC) on Superdex 200 Increase 10/200 GL columns (GE Healthcare) in Dulbecco’s phosphate-buffered saline (DPBS; Thermo Fisher Scientific). Proteins were analyzed by analytical SEC (Superdex 200 Increase 5/150 GL column, GE Healthcare). Thermal stability was measured by nano differential scanning fluorimetry (nanoDSF) on a Tycho NT.6 instrument (NanoTemper Technologies).

### Cell Lines

The L-428 cell line was purchased from the “German Collection of Microorganisms and Cell Cultures” (DSMZ, Leibniz Institute, Braunschweig, Germany). Cells were cultured in RPMI 1640 medium supplemented with GlutaMAX (Gibco, Thermo Fisher Scientific) and 10% fetal bovine serum (FBS; Gibco, Thermo Fisher Scientific). The Expi293F cell line was cultured in Expi293 Expression Medium (Thermo Fisher Scientific).

### Preparation of Peripheral Blood Mononuclear Cells From Whole Blood

Peripheral blood samples were collected from healthy donors (HDs) giving written informed consent according to a clinical protocol (“*In vitro* studies to establish new immunotherapies for AML and other hematological neoplasias”) approved by the Ethics Committee at the LMU Munich. Peripheral blood mononuclear cells (PBMCs) from HDs were separated from peripheral blood using the Histopaque-1077 Hybri-Max separating solution (Sigma-Aldrich) and Leucosep tubes (Greiner Bio-One) according to the manufacturer’s instructions. In brief, 15 ml of Histopaque solution was preloaded into a 50 ml Leucosep tube by centrifugation for 30 s at 1,000 g. The heparinized whole-blood samples were diluted with equal volumes of DPBS, and 30 ml of the diluted blood was added to the Leucosep tube. Tubes were centrifuged for 10 min at 1,000 g with the brakes off. The buffy coat containing PBMCs was collected, and cells were washed and re-suspended in RPMI 1640 containing 10% FBS for immediate use.

### Generation, Maturation, and Peptide-Loading of Monocyte-Derived DCs

Monocyte-derived DCs (moDCs) were generated within 3 days as previously described (41). Monocytes were enriched from PBMCs by plastic adherence in flat-bottom 6- or 12-well plates with surface treatment for maximum adhesion (Nunc, Thermo Fisher Scientific) at a concentration of 0.5–1 × 10^7^ cells/ml in VLE RPMI (Biochrom) supplemented with 1.5% human serum (HS, serum pool of AB positive adult males; Institute for Transfusion Medicine, Suhl, Germany)—hereafter termed DC medium. If required, non-adherent cells (NACs) were kept at 37°C and 5% CO_2_ until further use three days later. For microscopy experiments requiring pure monocytes, monocytes were isolated from PBMCs using the Classical Monocyte Isolation kit (Miltenyi Biotec) according to the manufacturer’s instructions. Next, cells were seeded in 24-well Nunc-plates and cultured for 48 h at 37°C and 5% CO_2_ in DC medium supplemented with 800 IU/ml of GM-CSF (Peprotech) and 580 IU/ml of IL-4 (Peprotech). These cells were then loaded with 200 nM antibody with or without flagellin fusion for 24 h, optionally pre-incubated for 30 min with a fourfold excess of αTLR5-IgA2 (Q2G4) or corresponding isotype control (T9C6, both InvivoGen). If moDCs were to be maintained at the immature stage (iDCs), cells were incubated with 800 IU/ml of GM-CSF, 580 IU/ml of IL-4, and 250 ng/ml of PGE_2_ (Sigma-Aldrich) for a further 24 h. To generate fully mature moDCs (mDCs), maturation was achieved within 20–24 h after addition of 800 IU/ml of GM-CSF, 580 IU/ml of IL-4, 250 ng/ml of PGE2, 2000 IU/ml of IL-1β (R&D Systems), 1,100 IU/ml of TNF-α (Peprotech), 5,000 IU/ml of IFN-γ (Peprotech), and 1 µg/ml of R848 (InvivoGen) (41, 42). For DC–T cell co-culture experiments, iDCs and mDCs were pulsed with the processed HLA-A*02:01-restricted peptides (CMV_NLV_, mNPM1_CLA_) for 1.5 h at 37°C.

### Surface Phenotyping of DCs and IL-6 Secretion

Immunofluorescent staining of DC surface antigens was performed using a panel of fluorescence-conjugated monoclonal antibodies and analyzed by flow cytometry: CD80 (BV510, 2D10), CD83 (PerCP-Cy5.5, HB15e), CD86 (APC, BU63), and HLA-DR (Pacific Blue, L243, all BioLegend). DCs could be separated from non-differentiated monocytes according to their higher FSC/SSC intensities. Mean fluorescence intensity (MFI) ratios were determined by dividing the MFI value measured for the antibody by that of the corresponding isotype control. In parallel, IL-6 secretion into the supernatant was quantified *via* cytometric bead array (BD Biosciences) according to the manufacturer’s instructions.

### Quantitative Analysis of Binding by Flow Cytometry

To determine equilibrium binding constants (*K*_D_; as an avidity measurement), L-428 cells were incubated with antibodies in concentrations ranging from 0.005 to 200 nM and stained with an αhuman IgG secondary antibody (FITC, HP6017, BioLegend). The assay was performed in triplicates, and data points were normalized to the maximum MFI and analyzed by non-linear regression using a one-site specific binding model.

To determine maximum binding, L-428 cells, iDCs or mDCs were incubated with the antibodies at a concentration of 200 nM followed by secondary antibody staining and MFI ratios were calculated.

### Quantitative Analysis of Binding by Surface Plasmon Resonance

For determining *K*_D_ values by surface plasmon resonance (SPR; Biacore X100, GE Healthcare), a CM5 chip (GE Healthcare) and the αhuman Fc capture (GE Healthcare) were used. The extracellular domain of CD40 was passed over the antibody-coated chip at concentrations ranging from 15.62 to 1,000 nM. *K*_D_ values were determined as the ratio of the association rate constant (*k*_on_) and the dissociation rate constant (*k*_off_).

### Interaction and Signaling Studies Using the hTLR5 Reporter Cell Line

HEK293-T cells were seeded in 24-well plates and transfected at 70% confluency the next day. 1 h before transfection, the medium was exchanged for 0.5 ml of OptiMEM medium (Gibco, Thermo Fisher Scientific) containing 5% FBS. 200 ng of human TLR5 plasmid was transfected using Lipofectamine 2000 (Invitrogen, Thermo Fisher Scientific) according to the manufacturer’s instructions (hTLR5-HEK293). At 22 h post-transfection, the medium was replaced with 1 ml of fresh DMEM medium supplemented with 5% FBS and further incubated for TLR5 expression (43). At 44 h post transfection, cells were incubated for 4 h with 10 nM/well of CD40^CMV^, αCD40.Flg^CMV^, αCD40.mFlg^CMV^, or Fc.Flg. Before the coincubation, all proteins were prepared briefly by sonication for 1 min at 4°C and power 5, to release monomeric flagellin, which is the active molecule binding to TLR5 (Branson Ultrasonics). Purified endotoxin-free native *Salmonella* Typhimurium full-length flagellin (100 ng/well) was used as a positive control protein to evaluate the TLR5-dependent activation in each experiment. IL-8 secretion in the supernatants of the activated cells was determined using an IL-8 OptEIA™ ELISA system (BD Biosciences), according to the manufacturer’s recommendations. Empty vector-transfected cells were used as control conditions for each experiment. Those conditions did not produce any significant elevation of IL-8 secretion over mock-coincubated with any of the tested proteins, indicating that the response was strictly TLR5-dependent.

### Expansion of CMV_NLV_ and mNPM1_CLA_ Peptide-Specific T Cells

To generate CMV_NLV_-specific T cells that recognize the pp65-derived CMV_495-503_ epitope (NLVPMVATV) in the context of HLA-A*02:01, PBMCs from an HLA-A*02:01^+^ and CMV^+^ donor were isolated. Mature DCs were produced as described above, pulsed with 1 µM CMV_NLV_ (JPT Peptide Technologies) for 90 min, then irradiated with 30 Gy (XStrahl RS225, XStrahl). Autologous CD8^+^ T cells were isolated from NACs using the CD8^+^ T Cell Isolation Kit (Miltenyi Biotec). T cells and pulsed DCs were co-cultivated at a T cell:DC ratio of 4:1 in RPMI 1640 containing 5% HS and 30 ng/ml of IL-21 (Peprotech) for 72 h. On days 3 and 6, co-cultures were expanded 1:1 by the addition of medium supplemented with 10 ng/ml of IL-15 and 10 ng/ml of IL-7 (both Peprotech). On day 9, CMV_NLV_-specific T cells were sorted on a FACSAria III cell sorter (BD Biosciences) by staining for CD8 (FITC, SK1, BioLegend) and with HLA-A*02:01-pCMV_NLV_ dextramer (PE, Immudex).

mNPM1_CLA_-specific T cells that recognize the mNPM1_288-296_ epitope (CLAVEEVSL) in the context of HLA-A*02:01 were generated as previously described by the transduction of CD8^+^ T cells with a mNPM1_CLA_-specific T cell receptor (TCR) (kindly provided by Dr. Marieke Griffioen, Leiden University) (44).

For expansion of specific T cells, PBMC feeders of two HLA-A*02:01^+^ and two HLA-A*02:01^−^ donors were mixed in equal amounts and pulsed with 1 µM CMV_NLV_ or mNPM1_CLA_ peptide (JPT Peptide Technologies) in X-VIVO 15 medium (Lonza) for 2 h at 37°C. After irradiation with 30 Gy, feeders were cultivated at a concentration of 2 × 10^6^ cells/ml with specific T cells at 0.4 × 10^6^ cells/ml in X-VIVO 15 containing 5% HS supplemented with 10 ng/ml of IL-7, 10 ng/ml of IL-15, and 0.5 µg/ml of PHA-L (Sigma-Aldrich) in a 6-well plate. After 3 days, cultures were fed by replacing half the volume of medium with fresh X-VIVO 15 containing 5% HS supplemented with 50 U/ml of IL-2 (Peprotech), 20 ng/ml of IL-7, and 20 ng/ml of IL-15. Using this medium, T cells were expanded every 3 days. Experiments were performed 9–21 days after expansion. Feeding was necessary every 14–21 days to re-challenge the cells with peptide.

### Internalization Assay by Structured Illumination Microscopy

To assess internalization of antibodies by DCs, moDCs differentiated from magnetically isolated monocytes were used. αCD40^CMV^ and the αHer2^CMV^ non-specific binding control were labeled with Alexa Fluor (AF)594 Labeling Kit (Thermo Fisher Scientific) according to the manufacturer’s instructions. iDCs and mDCs were incubated for 90 min at 4°C or 37°C with 200 nM labeled antibodies. For samples incubated at 4°C and the αHer2 controls at either temperature, membrane staining with an AF488-labeled αHLA-DR antibody was performed. Next, moDCs were transferred onto coverslips using a Shandon Cytospin 3 cytocentrifuge (Thermo Fisher Scientific). Fixation was performed with 4% paraformaldehyde (Sigma-Aldrich) followed by permeabilization with 0.1% Triton X-100 (Sigma-Aldrich). Lysosomes were stained using αLAMP1 (polyclonal, Novus Biologicals) or αEEA1 antibody (F.43.1, Thermo Fisher Scientific), which were detected using a secondary donkey αrabbit AF488 antibody (polyclonal, Thermo Fisher Scientific). Post-fixation took place with 4% paraformaldehyde followed by nuclear staining with 1 µg/ml of DAPI (Thermo Fisher Scientific). Finally, the coverslips were mounted onto glass slides using Vectashield (Vectorlabs).

3D structured illumination microscopy (SIM) acquisition was done on a Deltavision OMX V3 microscope (General Electric) equipped with a 100 × 1.4 oil immersion objective UPlanSApo (Olympus), 405, 488, and 593 nm diode lasers and Cascade II EMCCD cameras (Photometrics). Raw data were first reconstructed and corrected for color shifts with the provided software softWoRx 6.0 Beta 19 (unreleased). A custom-made macro in Fiji finalized the channel alignment and established composite TIFF stacks (45).

### Autologous DC–T Cell Co-Culture Using Non-Adherent Cells as the T Cell Source

DCs were washed prior to co-culturing with T cells to remove antibodies, maturation reagents and peptides. Peptide- or antibody-loaded iDCs and mDCs were incubated with NACs of an HLA-A*02:01^+^ and CMV^+^ donor in a 1:10 ratio in DC medium for 6 days. Dead cells were excluded as 7-AAD-positive cells (BioLegend) and the percentage of CMV_NLV_-specific CD8^+^ T cells was determined by staining CD8^+^ cells (FITC, SK1, BioLegend) with HLA-A*02:01-pCMV_NLV_ dextramer (PE, Immudex) and analyzed by flow cytometry.

### Allogeneic DC–T Cell Co-Culture Using Expanded CMV_NLV_- and mNPM1_CLA_-Specific T Cells

Peptide- or antibody-loaded iDCs and mDCs were cultivated with allogeneic expanded CMV_NLV_- and mNPM1_CLA_-specific T cells in a 1:5 ratio for 4–6 h in DC medium containing 25 µM monesin and 10 µg/ml of brefeldin A (both Sigma-Aldrich) at 37°C and 5% CO_2_. Viable Zombie Green-negative (BioLegend) cells were stained for CD8 (PerCP-eFluor710, SK1, eBioscience), subsequently fixed and permeabilized using the BD Cytofix/Cytoperm Kit (BD Biosciences) according to the manufacturer’s instructions followed by intracellular IFN-γ (PE, B27) and TNF-α (APC, Mab11, both BioLegend) staining. The percentage of cytokine-secreting CD8^+^ T cells was determined by flow cytometry.

### Flow Cytometry

Flow cytometry measurements were performed either on a Guava easyCyte 6HT flow cytometer (Merck Millipore) with data analyzed using GuavaSoft version 3.1.1 (Merck Millipore) or on a CytoFLEX LX flow cytometer (Beckmann Coulter) with FlowJo 10.6 (Tree Star Inc.).

### Data Plotting and Statistical Analysis

Data was analyzed using GraphPad Prism 8.0.2 (GraphPad Software). Differences in DC maturation, T cell activation and proliferation in comparing different donors were assessed using a Wilcoxon-signed rank test. For experiments including the hTLR5-HEK293 reporter cell line, an unpaired, two-tailed Student’s *t*-test was performed. Statistical significance was considered for *p*-values < 0.05 (*), < 0.01 (**), < 0.001 (***), and < 0.0001 (****).

## Results

### αCD40^CMV^ and αCD40.Flg^CMV^ are Pure and Stable Proteins That Bind to DCs

**Figure 1:**
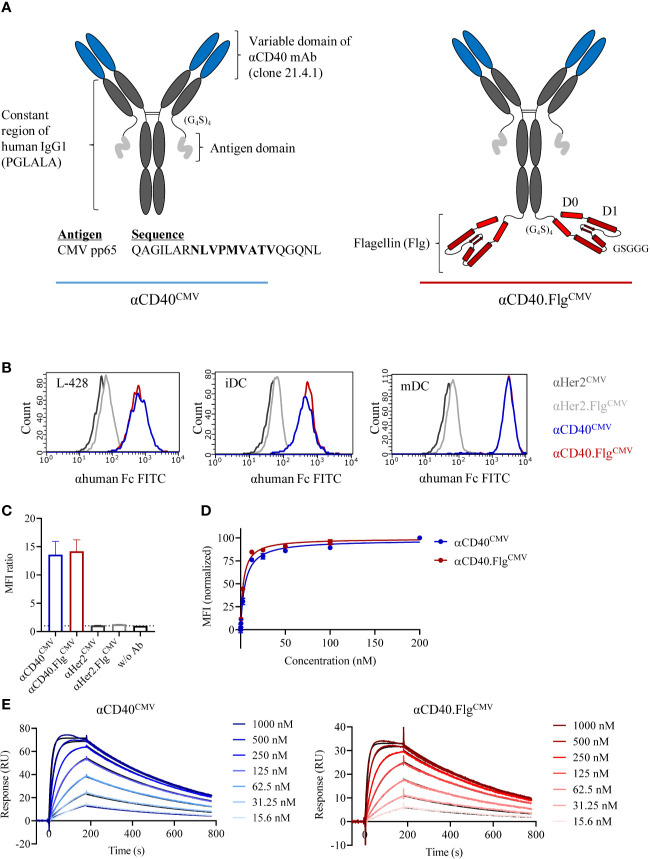
Modular composition and binding characteristics of αCD40^CMV^ and αCD40.Flg^CMV^. **(A)** Schematic drawing of αCD40^CMV^ (left) and αCD40.Flg^CMV^ (right). The CMV epitope sequence is shown in bold. PGLALA are amino acid mutations introduced for Fc silencing. **(B)** Histograms of αCD40^CMV^ and αCD40.Flg^CMV^ compared to the non-specific binding controls αHer2^CMV^ and αHer2.Flg^CMV^ at saturating concentrations analyzed by flow cytometry. Binding to L-428 cells, immature (iDCs), and mature (mDCs) monocyte-derived DCs (moDCs) was assessed. Histograms from one of three experiments are shown and are typical of the data. **(C)** Maximum binding of αCD40^CMV,^αCD40.Flg^CMV^ and the respective Her2 controls to iDCs from different donors, as measured by flow cytometry (*n* = 4). **(D)** Binding curves of αCD40^CMV^ and αCD40.Flg^CMV^ in L-428 cells as determined by flow cytometry (*n* = 3). **(E)** Representative SPR profiles for αCD40^CMV^ and αCD40.Flg^CMV^. An αhuman Fc antibody was coated onto a CM5 chip to immobilize αCD40 IgG1 antibodies. The extracellular domain of CD40 (the analyte) was injected and the concentration-dependent on- and off-rates were determined. Colored curves represent raw data and black curves fitted data. Data represent mean ± SEM.

To deliver immunogenic peptides specifically to CD40 on DCs, we first generated proof-of-principle constructs with a CMV-specific peptide as a model antigen. It consisted of a CMV peptide-coupled αCD40 antibody (αCD40^CMV^) with an Fc-silenced IgG1 backbone (**Figure 1A**). The variable regions are derived from the therapeutic IgG2-based αCD40 antibody CP-870,893 (clone 21.4.1) (37, 38). To combine DC targeting with TLR5 agonist-mediated stimulation, we fused the D0/D1 domain of *Salmonella* Typhimurium flagellin (Flg), structurally similar to the therapeutic molecule entolimod, to the C-termini of the heavy chains of the antibody portion using a flexible linker of four repeating polyglycine-serine (G_4_S) units (αCD40.Flg^CMV^). The peptide domain is connected to the C-termini of the light chains by a (G_4_S)_4_ linker and consists of a partial sequence of the CMV-specific pp65 protein including the HLA-A*02:01-restricted CMV_495–503_ epitope (NLVPMVATV, CMV_NLV_). Purified proteins were analyzed by analytical SEC (**Supplementary Figure S1A**) and protein stability was assessed by nanoDSF (**Supplementary Figure S1B**). Both αCD40^CMV^ and αCD40.Flg^CMV^ can be purified to homogeneity without noticeable aggregation and exhibit two melting transitions at around 72°C and 84°C, independent of the flagellin domain.

We evaluated the binding properties of the antibody constructs to APCs by flow cytometry. An antibody directed against Her2 served as a non-specific binding control since DCs are Her2 negative. No differences in MFI ratios for αCD40^CMV^ and αCD40.Flg^CMV^ were measured with either L-428 cells or moDCs. However, MFI ratios were higher on mDCs compared to iDCs due to upregulation of CD40 during the maturation process (**Figure 1B**). Further maximum binding analysis on iDCs from different donors confirmed that the flagellin domain neither impaired nor contributed to DC binding, suggesting that flagellin does not interfere with the CD40 binding domain (**Figure 1C**). Furthermore, apparent dissociation constants (*K*_D_) were determined by flow cytometry of L-428 cells (**Figure 1D**) and SPR analysis (**Figure 1E**). By flow cytometry, we measured affinities in the low nanomolar range (*K*_D_ αCD40^CMV^ = 4.9 ± 0.3 nM, αCD40.Flg^CMV^ = 3.0 ± 0.2 nM; for *n* = 3), whereas *K*_D_ values measured by SPR were higher due to monovalent binding (*K*_D_ αCD40^CMV^ = 27.4 ± 3.7 nM, αCD40.Flg^CMV^ = 21.8 ± 1.1 nM; for *n* = 3). Both experiments showed that the flagellin fusion did not substantially alter *K*_D_ values, again indicating no noticeable perturbation of CD40-binding functionality.

### αCD40^CMV^ Internalizes Into DCs Upon CD40 Binding

**Figure 2:**
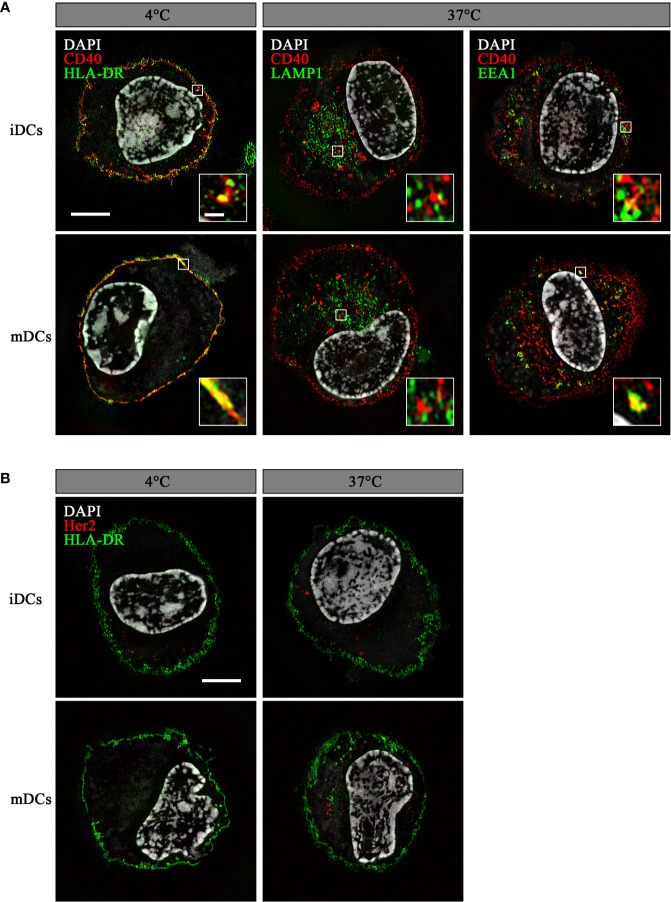
αCD40_CMV_ internalized into early endosomal compartments upon binding to CD40. Internalization of αCD40^CMV^-AF594 **(A)** or of the non-specific binding control αHer2^CMV^-AF594 **(B)** into iDCs and mDCs after incubation for 1.5 h at 4°C and 37°C. Co-staining with αHLA-DR-AF488 (membrane) was performed at 4°C and with αHer2^CMV^ at 37°C. αCD40^CMV^-treated DCs were stained with αLAMP1-AF488 (late endosomes) and αEEA1-AF488 (early endosomes) at 37°C. Internalization was visualized by structured illumination microscopy. Fluorescent signal within enlarged images is enhanced until saturation of the endosomal signals to better visualize co-localization. The scale bar represents 5 µm and in the enlarged images 0.5 µm.

To ensure endosomal processing of the attached peptide and efficient cross-presentation, DC-targeting antibodies need to internalize after binding their cognate receptors. Therefore, we analyzed the ability of DCs to internalize αCD40^CMV^ by fluorescence microscopy (**Figure 2**). For this, we incubated iDCs and mDCs with an αCD40^CMV^ antibody directly labeled with AF594. Specific internalization by targeting CD40 was compared to non-specific internalization using the αHer2^CMV^ control. At 4°C, no internalization occurred, as indicated by a co-localization with HLA-DR on the cell surface. At 37°C, specific internalization mediated by CD40 was detected within 1.5 h, suggesting a specific mechanism mediated by CD40. αCD40^CMV^ translocated specifically to EEA1^+^ early endosomal compartments, but not to LAMP1^+^ late endosomes (**Figure 2A**). This agrees with data reported for another αCD40 clone (S2C6) (12, 13). Only minimal non-specific internalization was visible using the αHer2^CMV^-AF594 control for all conditions (**Figure 2B**). We also observed no obvious difference between iDCs and mDCs.

### The Low αCD40^CMV^-Mediated Upregulation of Maturation Markers on iDCs Is Highly Amplified by Genetic Fusion With a Flagellin Domain

**Figure 3:**
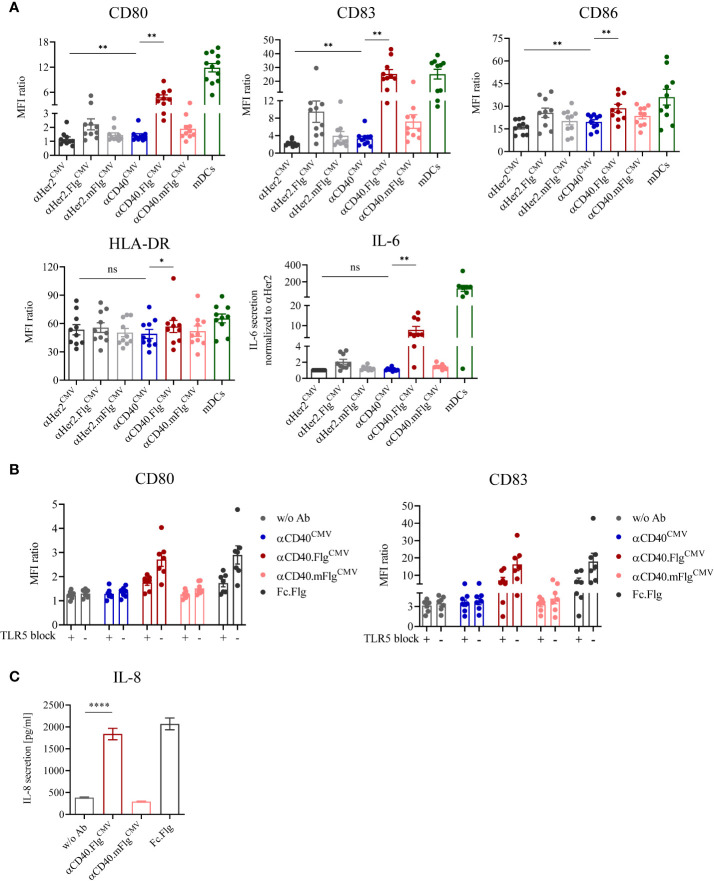
DC maturation induced by αCD40^CMV^ with low intrinsic agonistic activity was significantly boosted by the fusion of bacterial flagellin, which activates the DC specifically *via* interaction with TLR5. **(A)** Expression of surface maturation markers and secretion of IL-6 by iDCs as measured by flow cytometry and cytometric bead array, respectively. iDCs were stimulated with αCD40^CMV^, αCD40.Flg^CMV^, αCD40.mFlg^CMV^ or the respective αHer2 non-specific binding controls. TLR7/8-matured mDCs were included as positive controls (*n* = 10 donors). **(B)** Expression of surface maturation markers on iDCs in combination with TLR5 blocking prior to stimulation with CD40^CMV^, αCD40.Flg^CMV^, αCD40.mFlg^CMV^, Fc.Flg, or without antibody (w/o Ab) (*n* = 7 donors). **(C)** Secretion of IL-8 by HEK293-T cells transiently transfected with hTLR5 after incubation with αCD40.Flg^CMV^, αCD40.mFlg^CMV^ or Fc.Flg. The experiment was performed in biological replicates for each condition and measured in triplicates. For statistical analysis, a Wilcoxon-signed rank test **(A, B)** or unpaired, two-tailed Student’s *t*-test **(C)** were applied. Bars represent mean ± SEM. *p < 0.05, **p < 0.01, ****p < 0.0001, ns not significant.

The antibody clone used in this work was previously characterized to bind agonistically to CD40 in its initial IgG2 format (37, 38). Therefore, we expected that maturation of DCs is induced by the αCD40 antibody on its own and, if our approach works as anticipated, can be further enhanced by ligation of TLR5 by αCD40.Flg^CMV^. To investigate TLR5-specific effects, the mutations R90A and E114A (mFlg) were introduced to the flagellin domain, which have been individually or in combination with further mutations shown to drastically reduce flagellin binding to TLR5 (46–48). To test DC-maturating activities, we incubated iDCs for 24 h with αCD40^CMV^, αCD40.Flg^CMV^, αCD40.mFlg^CMV^ or the respective αHer2 controls. TLR7/8-maturated mDCs were included as a positive control (41, 42). Subsequently, we analyzed the DC surface markers CD80, CD83, CD86, and HLA-DR, as well as the secretion of IL-6 by flow cytometry as responses to the maturating stimuli (**Figure 3A**). αCD40^CMV^ itself led to a slight, but significant upregulation of most maturation markers compared to the αHer2 control, indicating a perceptible intrinsic agonistic activity. Treatment of iDCs with αCD40.Flg^CMV^, in contrast, caused a strong and significant upregulation of all maturation markers and IL-6 secretion compared to αCD40^CMV^. These effects were abolished by introducing the two crucial mutations into the flagellin portion.

To investigate whether the genetic fusion of αCD40^CMV^ and flagellin maintains functionalities of both fusion partners, we compared the activities of αCD40.Flg^CMV^ to co-administered Fc.Flg and αCD40^CMV^ (**Supplementary Figure S2A**). Even if a difference between the two variants was observed for the αHer2 control molecule, treatment with αCD40.Flg^CMV^ and also the combination of Fc.Flg and αCD40^CMV^ led to similar DC maturation states, as reflected by the increased expression of most of the surface markers. This indicates that fusion of flagellin to αCD40^CMV^ does not greatly affect its interplay with TLR5 and that neither antibody nor flagellin integrity is impaired.

To verify that the fused flagellin domain activates DCs specifically *via* TLR5, we studied the activation potential of αCD40.Flg^CMV^ with and without TLR5-receptor blockage and the induction of TLR5-specific downstream signaling. First, DC maturation triggered by flagellin was investigated in the presence of an αTLR5-blocking and neutralizing IgA antibody. To this end, iDCs were pre-incubated with either the blocking antibody or the isotype control followed by the addition of αCD40^CMV^, αCD40.Flg^CMV^, αCD40.mFlg^CMV^, or Fc.Flg for 24 h. The flagellin-induced upregulation of DC maturation markers was greatly diminished by the presence of the αTLR5 antibody, but not by the isotype control (**Figure 3B**). Therefore, the TLR5 interaction is necessary for stimulation of DC maturation by αCD40.Flg^CMV^ and Fc.Flg. Next, the activation of TLR5-specific downstream signaling processes by the proprietary flagellin fusion molecules was studied. For this purpose, a hTLR5-HEK293 reporter cell line, transiently transfected with full-length human TLR5, was incubated with abovementioned fusion proteins and TLR5-specific signal transduction was quantitated based on IL-8 secretion. As expected, αCD40.Flg^CMV^ and Fc.Flg induced TLR5 signaling as shown by similar levels of IL-8 secretion, whereas αCD40.mFlg^CMV^ did not (**Figure 3C**).

Taken together, the results show that αCD40.Flg^CMV^ significantly enhanced DC maturation compared to αCD40^CMV^ by the specific interaction of flagellin with TLR5.

### CMV-Derived Peptides Targeted to DCs *via* αCD40 are Efficiently Cross-Presented and Induce Activation and Proliferation of Antigen-Specific T Cells in DC–T Cell Co-Cultures

**Figure 4:**
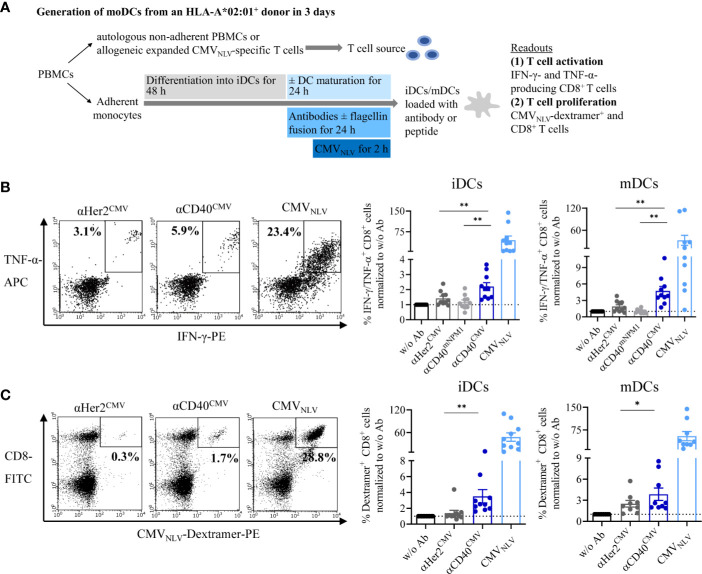
αCD40^CMV^ loaded DCs efficiently cross-presented the peptides on the surface and induced activation and proliferation of antigen-responsive T cells. **(A)** Co-cultures of iDCs/mDCs and allogeneic CMV_NLV_-specific T cells or with autologous NACs from HLA-A*02:01^+^ donors with a preceding CMV infection. DCs were pre-incubated with different antibody constructs or with peptide controls. In the allogeneic setting, T cell activation was analysed by counting IFN-γ- and TNF-α-producing CD8^+^ cells. In the autologous setting, T cell proliferation was determined by CMV_NLV_-specific dextramer staining of CD8^+^ T cells. **(B)** Co-culture of allogeneic CMV_NLV_-specific T cells and HLA-A*02:01^+^ iDCs or mDCs. The DCs were pre-incubated with αHer2^CMV^, αCD40^CMV^, αCD40 conjugated to a control peptide (αCD40^mNPM1^) or with the processed CMV_NLV_ peptide. As a readout, T cell activation was measured by flow cytometry (*n* = 10 donors). **(C)** Co-culture of autologous NACs with iDCs and mDCs pre-loaded with αHer2^CMV^, αCD40^CMV^ and the processed CMV_NLV_ peptide. As readout, T cell proliferation was analysed by flow cytometry (*n* = 10 donors). T cell activation and proliferation are normalized to w/o Ab for each donor, respectively. Bars represent mean ± SEM. For statistical analysis, a Wilcoxon-signed rank test was applied. *p < 0.05, **p < 0.01.

We addressed the question of whether the CMV-derived peptide fused to αCD40 is correctly processed into the epitope sequence NLVPMVATV (CMV_NLV_) and subsequently cross-presented on the DC surface *via* MHC-I. The interaction with peptide-responsive T cells was validated based on T cell activation and proliferation in autologous and allogeneic DC–T cell co-culture experiments (**Figure 4A**). Prior to co-culturing with T cells, iDCs or mDCs from HLA-A*02:01^+^ donors were pre-loaded with either αCD40^CMV^ or αHer2^CMV^. To rule out the possibility of T cell activation triggered by αCD40-mediated DC maturation and to confirm antigen specificity, an αCD40 antibody coupled with a non-stimulating peptide (αCD40^mNPM1^, vide infra) was used as a control in the allogeneic setting. T cell functionality was validated by pulsing the DCs with the already processed CMV_NLV_ peptide.

First, cross-presentation and T cell interaction were investigated by incubating pre-loaded DCs with allogeneic and expanded CMV_NLV_-specific T cells (**Figure 4B**). After co-culture for 4 h, T cell activation was measured by intracellular IFN-γ and TNF-α staining of CD8^+^ T cells. Independently of the maturation state, αCD40^CMV^-loaded DCs elicited significantly higher T cell activation compared to αCD40^mNPM1^ and αHer2^CMV^ controls. As expected, a high level of secretion of pro-inflammatory cytokines was achieved with CMV_NLV_ peptide-pulsed DCs that do not require internalization, processing and cross-presentation. Next, we examined, whether T cells were stimulated sufficiently to proliferate after encountering CMV_NLV_-presenting DCs (**Figure 4C**). To this end, autologous DCs from an HLA-A*02:01^+^ donor with a preceding CMV infection were cultivated together with the non-adherent PBMC fraction (NACs) as a source of T cells. After 6 days of culture, CMV_NLV_-specific dextramer staining was performed to determine the percentage of expanded CMV_NLV_-responsive CD8^+^ T cells. In line with the results obtained for allogeneic co-cultures, loading of iDCs and mDCs with αCD40^CMV^ was accompanied by significantly greater CMV_NLV_-specific T cell proliferation compared to control molecules. T cell proliferation was at the highest level after interaction with CMV_NLV_-loaded DCs.

In summary, targeting CMV peptides to DCs *via* αCD40 leads to antigen processing into the correct epitope and cross-presentation and induces activation and proliferation of antigen-specific T cells. This validates the targeting approach and that the design affords functional constructs.

### Incubation of DCs With αCD40.Flg^CMV^ Elicits a Significantly Higher CMV_NLV_-Specific T Cell Activation and Proliferation Compared to αCD40^CMV^ in DC–T Cell Co-Cultures

**Figure 5:**
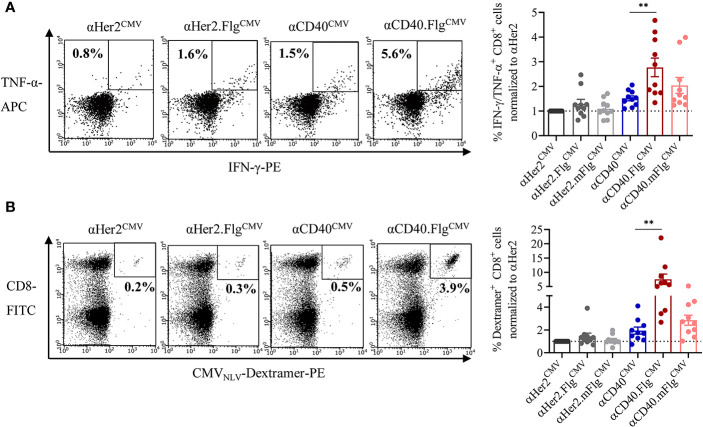
Flagellin fusion significantly enhanced CMV-specific T cell activation and proliferation. **(A)** Allogeneic DC–T cell co-culture of expanded and pre-activated CMV_NLV_-specific T cells and HLA-A*02:01^+^ iDCs. DCs were pre-incubated with αHer2.Flg^CMV^, αCD40.Flg^CMV^ and controls with mutated or no flagellin. As readout for T cell activation, IFN-γ-and TNF-α-producing CD8^+^ T cells was counted by flow cytometry (*n* = 10 donors). **(B)** iDCs of an HLA-A*02:01^+^ and CMV^+^ donor were pre-incubated with αCD40^CMV^, αCD40.Flg^CMV^, and αCD40.mFlg^CMV^ or the respective αHer2 controls and subsequently co-cultivated with autologous NACs. As a readout for T cell proliferation, CMV_NLV_-specific dextramer staining was performed and analyzed by flow cytometry (*n* = 10 donors). T cell activation and proliferation are normalized to the αHer2 control for each donor, respectively. Bars represent mean ± SEM. For statistical analysis, a Wilcoxon-signed rank test was applied. **p < 0.01.

Having validated the cross-presentation approach, we further analyzed whether the maturating activity of flagellin on DCs enhances cross-presentation and the T cell response. Accordingly, co-culture experiments were performed with allogeneic CMV_NLV_-responsive T cells or autologous NACs and iDCs that were pre-loaded with αCD40^CMV^, αCD40.Flg^CMV^, αCD40.mFlg^CMV^, or the respective αHer2 controls. Fusion of flagellin to αCD40 elicited a significantly higher level of IFN-γ and TNF-α secretion of antigen-responsive allogeneic T cells compared to the control that does not ligate TLR5 (**Figure 5A**). Moreover, loading of DCs with αCD40.Flg^CMV^ significantly increased the percentage of CMV_NLV_-specific CD8^+^ T cells in co-cultures with autologous NACs in comparison to αCD40^CMV^ (**Figure 5B**). No difference in T cell proliferation was measured between αCD40.Flg^CMV^ and the co-administered Fc.Flg and αCD40^CMV^, highlighting that coupling of the activating flagellin domain to the DC-targeting αCD40 antibody does not alter its functionality (**Supplementary Figure S2B**). In all experiments, mutations in the TLR5-binding region (αCD40.mFlg^CMV^) abolished benefits attributed to the flagellin domain. Conclusively, the delivery of antigens to DCs by a CD40-targeting antibody and the simultaneous activation by flagellin shows a clear advantage over αCD40^CMV^, not only in terms of DC maturation but also with respect to T cell activation and proliferation.

### Flagellin Fusion Also Enhances the Activation of mNPM1-Specific T cells in DC–T Cell Co-Cultures

To replicate these results of successfully enhanced T cell activation by flagellin fusion proteins in the AML setting, we exchanged the model antigen CMV_NLV_ for an AML-specific neoantigen. Accordingly, we fused the C-terminal sequence of the mutated NPM1 (mNPM1)-derived extended protein as antigen domain to the CD40-targeting antibody (αCD40^mNPM1^). The protein results from a frameshift mutation and includes the mNPM1_288-296_ neoepitope (CLAVEEVSL, mNPM1_CLA_), characteristic for AML (44). In a second step, the αCD40.Flg^mNPM1^ construct was generated to explore the effect of TLR5 ligation on the efficiency of induced specific T cell responses.

**Figure 6:**
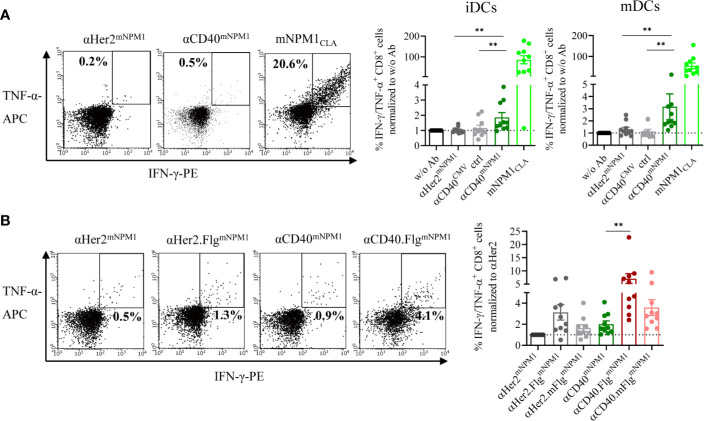
mNPM1-specific peptides fused to αCD40 were efficiently cross-presented on the DC surface and induced activation of mNPM1-responsive T cells that was significantly enhanced by TLR5 ligation. **(A)** Allogeneic DC–T cell co-culture of mNPM1_CLA_-specific T cells and HLA-A*02:01^+^ iDCs. DCs were pre-incubated with αHer2^mNPM1^, αCD40^mNPM1^, αCD40 conjugated to a control peptide (αCD40^CMV^) or with the processed mNPM1_CLA_ peptide. As a readout for T cell activation, IFN-γ- and TNF-α- producing CD8^+^ T cells were counted by flow cytometry (*n* = 10 donors). **(B)** Allogeneic DC–T cell co-culture of mNPM1_CLA_-specific T cells and HLA-A*02:01^+^ iDCs. DCs were pre-incubated with αCD40^mNPM1^, αCD40.Flg^mNPM1^, αCD40.mFlg^mNPM1^ or the respective αHer2 controls. T cell activation was measured by flow cytometry (*n* = 10 donors) and normalized to w/o Ab or to the αHer2 control for each donor, respectively. Bars represent mean ± SEM. For statistical analysis, a Wilcoxon-signed rank test was applied. **p < 0.01.

First, we investigated whether iDCs and mDCs incubated with αCD40^mNPM1^ cross-present the processed mNPM1_CLA_ on their surface and activate peptide-responsive T cells. For target cells, we used expanded allogeneic T cells transduced with an mNPM1_CLA_-specific TCR generated by Lee et al. that were shown to recognize HLA-A*02:01^+^ and mNPM1^+^ AML cell lines and primary patient cells (44). We co-cultured those T cells with DCs that had been pre-incubated with αHer2^mNPM1^, αCD40^mNPM1^ or αCD40 conjugated with a non-stimulating control peptide (αCD40^CMV^). T cell functionality was validated using mNPM1_CLA_ peptide-pulsed DCs. αCD40^mNPM1^-loaded iDCs and mDCs elicited significantly greater T cell activation compared to those treated with αCD40 and αHer2 controls (**Figure 6A**). A high level of cytokine secretion was observed for the mNPM1_CLA_ peptide control, further confirming the specificity of the T cells. Finally, we investigated whether simultaneous TLR5 activation by αCD40.Flg^mNPM1^ favors a T cell response as previously observed in the CMV setting. In fact, in co-culture experiments with mNPM1_CLA_-responsive T cells, iDCs loaded with αCD40.Flg^mNPM1^ significantly increased the percentage of IFN-γ- and TNF-α-secreting CD8^+^ T cells compared to αCD40^mNPM1^ (**Figure 6B**).

These results demonstrate that mNPM1 peptides targeted to DCs by an αCD40^mNPM1^ antibody were processed and cross-presented efficiently on MHC-I molecules, thereby activating antigen-specific CD8^+^ T cells. Importantly, DC stimulation by TLR5 ligation was able to boost the T cell response. Thus, the proof-of-principle based on CMV was replicated in an AML setting with αCD40^mNPM1^ and αCD40.Flg^mNPM1^ showing that this approach has the potential to be developed into a therapy for AML.

## Discussion

The aim of this study was to generate a multifunctional antibody construct to co-deliver an antigen and adjuvant to CD40^+^ DCs for the induction of a peptide-specific T cell response. This construct was designed to combine the potential advantages of *in vivo* DC vaccination with those of targeted delivery of TLR agonists, in this case a protein agonist of TLR5, which is easily amenable to protein fusion constructs.

Our *in vitro* experiments demonstrated that αCD40^CMV^ presenting CMV as a model antigen for proof-of-principle studies binds specifically to CD40. However, the simple fusion construct induces only a low upregulation of DC maturation markers upon binding. In contrast, the enhanced fusion construct containing a flagellin domain as TLR5 agonist provided a much higher activation potential of DCs and a better activation of antigen-specific T cells. The variable regions of the αCD40 portion used in this study are derived from the IgG2-type αCD40 clone CP-870,893 (selicrelumab) with high agonistic activity. Severe treatment-related adverse events such as cytokine release syndrome and hepatotoxicity have been reported for the parental antibody, which has been investigated previously as a therapy against advanced solid tumors, limiting the dosage of treatment (37, 49). In addition, selicrelumab also induced chronic B cell activation associated with diminished circulating T cell numbers. This effect potentially resulted from activation-induced cell death as observed for other agonistic αCD40 antibodies along with T cell tolerance (50–52). This side effect might be particularly undesirable if those antibodies are to be used in the context of DC vaccines and limit their efficacy. In contrast, in the Fc-silenced IgG1 format as used in our assays, the αCD40 antibody only exhibits a low activating potential. This is consistent with the data from White et al. and Dahan et al. that indicate that the potency of agonistic CD40 antibodies is not only dependent on the epitope recognized, but can be influenced by different hinge regions and the level of Fc-mediated crosslinking (53, 54). Furthermore, we observed by structured illumination microscopy that αCD40^CMV^ internalized into early endosomal compartments after binding, which is in line with published data for a different αCD40 clone (12, 13). Chatterjee et al. have shown that the beneficial effect of antigen-delivery to CD40 on MHC-I cross-presentation and CD8^+^ T cell responses is predominantly promoted by the intracellular trafficking pathway and not by its activating potential (12). Thus, concerning the possibility of adverse events and impaired T cell function, our αCD40 Fc-silenced IgG1 antibody should combine the advantage of low intrinsic agonistic activity with the benefit of targeting of antigens to CD40 and their intracellular destination.

Since the low αCD40-mediated DC activation is likely to be insufficient for lasting T cell responses, a TLR agonist was included in the molecule as an adjuvant. We chose to target TLR5 since flagellin and its derivatives have been successfully used as vaccine adjuvants and have been shown to induce potent anti-viral and anti-tumor immune responses in animal studies and clinical trials (55–61). Vicente-Suarez et al. reported the ability of flagellin to convert tolerogenic DCs into activating APCs that preferentially induce Th1 responses in pre-clinical models (62). We selected a truncated version of flagellin similar to the clinically advanced molecule entolimod that retains the essential terminal TLR5-binding domains (63). In contrast to other TLR agonists, entolimod exhibited a unique and beneficial safety profile due to the restricted pattern of TLR5 expression in distinct organs and the nature of cytokines induced following TLR5 stimulation. In our experiments, the fusion of the truncated flagellin domain to αCD40^CMV^ was functional, upregulated maturation markers on DCs *via* TLR5 activation, and was much more effective than the simple αCD40 construct.

Autologous and allogeneic DC–T cell co-culture experiments demonstrated that αCD40^CMV^-loaded DCs effectively cross-presented CMV-derived peptides on their surfaces and interacted with CMV_NLV_-responsive T cells. This effect was significantly enhanced if T cells were cultivated with TLR5-stimulated DCs that had been pre-incubated with αCD40.Flg^CMV^. When we compared the immuno-stimulatory capacity of αCD40.Flg^CMV^ with separate administration of αCD40^CMV^ and an Fc-fused variant of flagellin, no clear difference was detected. Thus, there was no benefit in specific targeting *in vitro* under optimal conditions, which was an outcome to be expected. However, targeted delivery of TLR agonists in the context of protein fusion is likely of higher relevance *in vivo* minimizing dilution and buffering effects and potentially permitting lower dosages. As potential side effects at therapeutically relevant doses need to be investigated *in vivo*, mouse studies are indicated to support our hypothesis of reduced side effects and enhanced therapeutic efficacy. Still, *in vitro* data showed that the structural integrity of both αCD40 and flagellin is unaffected by their genetic fusing. This was further confirmed by binding studies and thermal unfolding measurements, which revealed that αCD40^CMV^ and αCD40.Flg^CMV^ have the same maximum binding and melting behavior. This is an important and novel finding, since other sites of genetic fusion or chemical modification of the TLR agonist have been shown to affect its physicochemical protein properties. In a similar approach, Kreutz et al. developed an antibody-antigen-adjuvant conjugate consisting of an αDec205-specific antibody conjugated *via* sulfo-SMCC linkers to the model antigen ovalbumin and CpG oligodeoxynucleotides. They observed that the CpG-conjugate was more potent at inducing cytotoxic T cell responses compared to the separate components when co-administered *in vivo*. Nevertheless, they encountered the problem that antibody binding and uptake was altered by the fusion with CpG allowing the delivery of both antigen and adjuvant to cells to be partially independent of the DC-targeting antibody (32). This was not observed for our antibody, probably due to its site-specific C-terminal fusion of the TLR agonist to the heavy chains with linkers of adequate length.

The multifunctional antibody construct was further validated using a neoantigen-derived antigen domain, making this the first study to investigate neoantigen delivery to a DC-targeting antibody construct. Applying neoantigens in a vaccination approach is exceptionally interesting as they arise from tumor-specific mutations and reactive T cells have not undergone clonal deletion. In the case of AML, neoantigens derived from NPM1 mutations are promising targets, as they occur in 30–35% of all AML patients and thus cover a broad spectrum of patients. Recently, Van der Lee et al. identified the mNPM1-derived HLA-A*02:01-specific neoepitope CLAVEEVSL among others by mass spectrometry analysis (44). Specific T cells were found in healthy individuals and a TCR with high reactivity was isolated. As a therapeutic approach, TCR-transduced T cells elicited anti-tumor efficacy in a mouse model and were able to recognize and kill mNPM1^+^ AML cell lines and primary patient samples *in vitro*. We generated DC-targeting antibody constructs with mNPM1 as an antigen domain, which is the first investigation of mNPM1 in a DC vaccination concept. In our experiments, DCs loaded with αCD40^mNPM1^, αCD40.Flg^mNPM1^ or with the processed mNPM1_CLA_ peptide potently activated mNPM1_CLA_-specific T cell responses. Further experiments are required to evaluate whether our molecules are able to expand the low abundance of mNPM1-specific T cells present in AML patients.

We demonstrated that the multifunctional antibody constructs αCD40.Flg^CMV^ and αCD40.Flg^mNPM1^ are highly potent activators of peptide-specific T cells. In particular, targeting mNPM1 to DCs is of utmost therapeutic significance. The *in vitro* experiments performed in this study show promising initial results for the development of αCD40.Flg^mNPM1^ as an *in vivo* DC vaccine strategy for AML. Future studies should examine the anti-tumor effects of this molecule as monotherapy and in combination with potential synergistic immunotherapies in AML xenograft models (64). The conjunction of neoepitope-based vaccines with immune checkpoint inhibition has resulted in sustained progression-free survival of cancer patients in clinical trials (4, 5). In addition, several clinical trials have explored the potential of post-transfer vaccination to enhance the clinical efficacy of adoptively transferred T cells expressing a TCR specific for an intracellular tumor antigen (65, 66). In line with this, combining αCD40.Flg^mNPM1^ with TCR gene therapy, in particular with mNPM1-specific T cells that recognize primary AML cells, would potentially show great promise to expand and enhance the efficiency of transferred T cells *in vivo* and to generate long-lasting responses (44).

Given the encouraging results of the molecule format in both the CMV and the mNPM1 setting, it is noteworthy, that these constructs could also serve as a vaccination platform to be potentially applied to any other (tumor) peptide as antigen domain.

## Data Availability Statement

The original contributions presented in the study are included in the article/**Supplementary Material**. Further inquiries can be directed to the corresponding authors.

## Ethics Statement

The studies involving human participants were reviewed and approved by Ethics Committee at the LMU Munich. The patients/participants provided their written informed consent to participate in this study.

## Author Contributions

SS, KD, NF, K-PH, and MS conceived and designed the experiments. SS generated and characterized the molecules, performed DC-based assays, and analyzed experiments. K-PH and ST designed the Flg fusion domain and ST helped interpreting the data. AL generated CMV-specific T cells and expanded peptide-specific T cells. AM performed microscopy studies under supervision of HL. CJ, MH, and LP designed, conducted, analyzed, and interpreted signaling experiments. CJ acquired funding. MR introduced dendritic cell cultivation techniques. MG provided mNPM1-specific T cells. SS designed the figures and wrote the manuscript with contribution of GH, MS, K-PH, NF, and ST. K-PH and MS supervised the project. All authors contributed to the article and approved the submitted version.

## Funding

This work was funded by German Research Council (DFG) provided within the Sonderforschungsbereich SFB1243 (MS, K-PH, and HL) and by the Bavarian Elite Graduate Training Network “i-Target” (SS, AL, ST, and MR). K-PH and ST were further supported by the Marie-Sklodowska-Curie Training Network “Immutrain” funded by the H2020 Program of the European Union (Grant 641549). MS was also supported by the Wilhelm Sander Stiftung (project number 2018.087.1). The lab of CJ was supported by the Deutsche Forschungsgemeinschaft (DFG), Sonderforschungsbereich SFB900 (Center grant CRC900), and project B6 and the German Center of Infection Research, DZIF, site Munich, project number 06.809. The production of mNPM1 TCR-transduced CD8^+^ T cells was supported by Miltenyi Biotec B.V. & Co. KG (Bergisch Gladbach, Germany), Health Holland project LSHM19120 and the Dutch Cancer Society project 11867.

## Conflict of Interest

The authors declare that the research was conducted in the absence of any commercial or financial relationships that could be construed as a potential conflict of interest.
