# Supplementary material for: Fusion of Bacterial Flagellin to a Dendritic Cell-Targeting αCD40 Antibody Construct Coupled With Viral or Leukemia-Specific Antigens Enhances Dendritic Cell Maturation and Activates Peptide-Responsive T Cells
Source: Front Immunol. 2020 Nov 12;11:602802. doi: 10.3389/fimmu.2020.602802 (PMC7689061; doi:10.3389/fimmu.2020.602802)
Supplement: Supplementary file 1 [file DataSheet_1.pdf]

Supplementary Material

1.1 Supplementary Figures

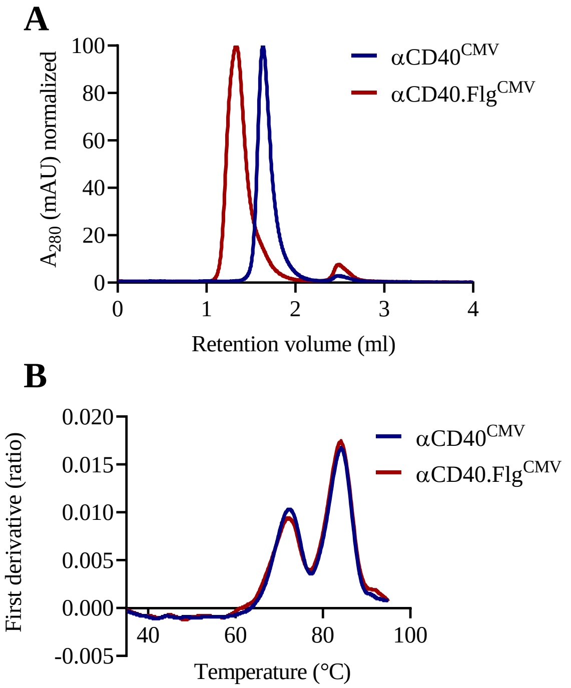

***T*m values:**

**Molecule**	***T*m (1)**	***T*m (2)**
αCD40^{CMV}	72.3°C	84.2°C
αCD40.Flg^{CMV}	72.1°C	84.1°C

Supplementary Figure S1: Expression, purity and stability of multifunctional antibody construct.

(A) Analytical size-exclusion chromatography of purified αCD40^{CMV} and αCD40.Flg^{CMV} using a Superdex 200 Increase 5/150 GL column. **(B)** Thermal stability of αCD40^{CMV} and αCD40.Flg^{CMV} as determined by nano differential scanning fluorimetry. Melting temperatures T_m are indicated in the table.

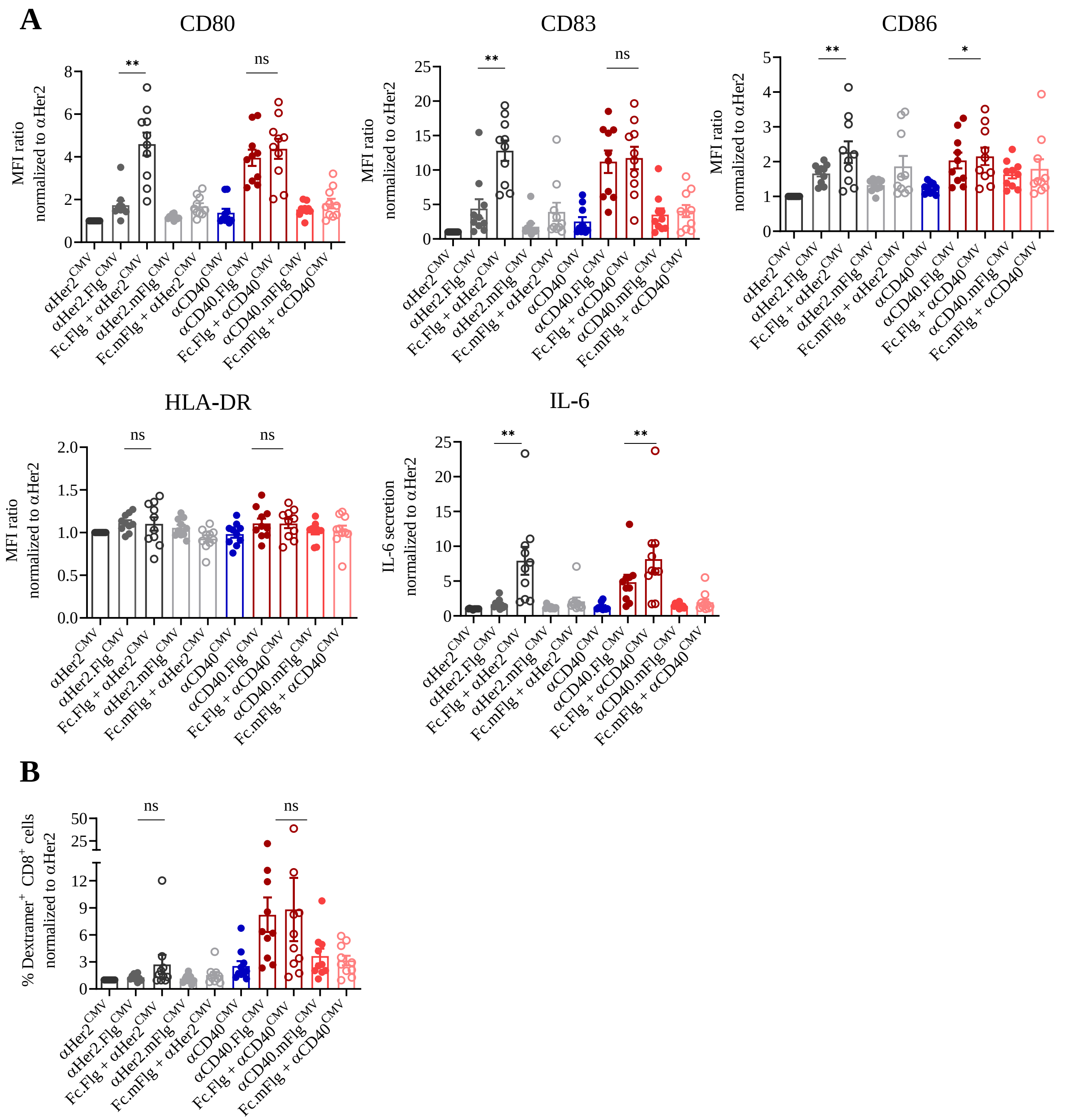

Supplementary Figure S2: Genetic flagellin fusion in αCD40.Flg^{CMV} and co-administration of αCD40^{CMV} with Fc.Flg similarly activated iDCs and elicited T-cell proliferation in co-culture experiments

(A) Expression of surface maturation markers and secretion of IL-6 by iDCs measured by flow cytometry and cytometric bead array, respectively. iDCs were loaded with αCD40.Flg^{CMV} or with a combination of αCD40^{CMV} ± Fc.Flg or the respective controls (*n* = 10 donors). **(B)** Proliferation of CMV$_{NLV}$-specific T cells of a HLA-A*02:01^{+} and CMV^{+} donor after stimulation with iDCs that were pre-incubated with antibody–flagellin fusion molecules or with the combination of αCD40^{CMV}/αHer2^{CMV} ± Fc.Flg/Fc.mFlg (*n* = 10 donors). As a readout for T-cell proliferation,

CMV_{NLV}-specific dextramer staining was performed and analyzed by flow cytometry. Data are normalized to the αHer2 control for each donor, respectively. Bars represent means ± SEM. For statistical analysis, a Wilcoxon-signed rank test was applied.
